# Dose-associated changes in safety and efficacy parameters observed in a 24-week maintenance trial of olanzapine long-acting injection in patients with schizophrenia

**DOI:** 10.1186/1471-244X-11-28

**Published:** 2011-02-15

**Authors:** Angela L Hill, Bin Sun, Jamie L Karagianis, Susan B Watson, David P McDonnell

**Affiliations:** 1 Lilly Research Laboratories, Eli Lilly and Company, Indianapolis, Indiana, 46285, USA; 2 Eli Lilly Canada Inc., 3650 Danforth Avenue, Toronto, Ontario M1N 2E8, Canada

## Abstract

**Background:**

In a recently published 24-week maintenance study of olanzapine long-acting injection (LAI) in schizophrenia (Kane et al., 2010), apparent dose-associated changes were noted in both efficacy and safety parameters. To help clinicians balance safety and efficacy when choosing a dose of olanzapine LAI, we further studied these changes.

**Methods:**

Outpatients with schizophrenia who had maintained stability on open-label oral olanzapine for 4 to 8 weeks were randomly assigned to "low" (150 mg/2 weeks; N = 140), "medium" (405 mg/4 weeks; N = 318), or "high" (300 mg/2 weeks; N = 141) dosages of olanzapine LAI for 24 weeks. Potential relationships between dose and several safety or efficacy measures were examined via regression analysis, the Jonckheere-Terpstra test (continuous data), or the Cochran-Armitage test (categorical data).

**Results:**

Safety parameters statistically significantly related to dose were mean weight change (low: +0.67 [SD = 4.38], medium: +0.89 [SD = 3.87], high: +1.70 [SD = 4.14] kg, p = .024; effect size [ES] = 0.264 high vs. low dose), mean change in prolactin (low: -5.61 [SD = 12.49], medium: -2.76 [SD = 19.02]), high: +3.58 [SD = 33.78] μg/L, p = .001; ES = 0.410 high vs. low dose), fasting triglycerides change from normal at baseline to high (low: 3.2%, medium: 6.0%, high: 18.9%, p = .001; NNT = 7 high vs. low dose) and fasting high-density lipoprotein cholesterol change from normal at baseline to low (low: 13.8%, medium: 19.6%, high: 30.7%, p = .019; NNT = 6 high vs. low dose). Efficacy measures significantly related to dose included Positive and Negative Syndrome Scale total score mean change (low: +2.66 [SD = 14.95], medium: -0.09 [SD = 13.47], high: -2.19 [SD = 13.11], p <.01; ES = 0.356 high vs. low dose), relapse rate (low: 16%, medium: 10%, high: 5%, p = .003; NNT = 9 high vs. low dose), all-cause discontinuation rate (low: 36%, medium: 30%, high: 24%, p = .037; NNT = 9 high vs. low dose), and rate of discontinuation due to efficacy-related reasons (low: 20%, medium: 14%, high: 6%, p <.001). Time to all-cause discontinuation (p = .035) and time to relapse (p = .005) were also significantly related to dose.

**Conclusions:**

Analyses of several safety and efficacy parameters revealed significant associations with dose of olanzapine LAI, with the highest dose generally showing greater efficacy as well as greater adverse changes in metabolic safety measures. When considering olanzapine LAI, as with all antipsychotics, it is important to carefully consider the potential benefits and risks for an individual patient.

**Trial Registration:**

ClinicalTrials.gov: NCT00088491

## Background

Olanzapine long-acting injection (LAI) has been studied in both the short-term [1] and long-term [2] treatment of schizophrenia. In a 24-week maintenance study of olanzapine LAI, apparent dose-associated changes were observed for both safety and efficacy parameters [2]. Some previous studies in patients treated with oral olanzapine have found an association between olanzapine plasma concentrations and changes in some metabolic parameters [3,4], as well as differences in changes in efficacy and safety parameters in a sample including patients treated with doses greater than 20 mg/day [5]. Considering these reports, the observed dose-associated changes were not completely unexpected and warranted further investigation. Reports of dose-associated changes involving other depot antipsychotics are sparse. In older, typical depots such as haloperidone decanoate, dose-related extrapyramidal symptoms (EPS) have long been observed [6]. Among atypical depots, risperidone long-acting injection has been reported as having dose-related changes for both weight and EPS, similar to that seen with oral risperidone [7]. Based on a limited number of studies, paliperidone palmitate may also have dose-related changes for weight, EPS, and prolactin [8].

Although the US prescribing information for olanzapine LAI [9] provides dosing recommendations based on the desired or target oral olanzapine dose, there is currently little information available to help clinicians balance safety and efficacy when selecting a dose. To that end, we conducted a further investigation of the dose-associated changes observed in the 24-week maintenance study mentioned above [2].

This article reports the results of post hoc analyses of a 24-week maintenance study of olanzapine LAI [2] examining the potential association between olanzapine LAI dose and several safety and efficacy parameters of clinical interest. Based on inspection of results previously reported [2], we hypothesized that significant dose-associated changes would be identified for both safety and efficacy parameters in this olanzapine LAI clinical trial.

## Method

A complete description of the clinical trial including the study design and patient population are found in the primary publication [2]. We provide only the methodological details pertinent to the post hoc analysis reported here. The study was conducted in accordance with the ethical principles set forth in the Declaration of Helsinki, good clinical practices (GCPs), and applicable laws and regulations. For each investigational site, ethical review boards provided written approval of the study protocol and the informed consent document.

### Patients

All patients from the 24-week maintenance study [2] who had been randomly assigned to 1 of 3 therapeutic doses of olanzapine LAI (N = 599) were included in these analyses. Patients were 18 to 75 years of age with a diagnosis of schizophrenia according to the DSM-IV or DSM-IV-TR.

### Measures

Safety/tolerability measures investigated included incidence of unsolicited treatment-emergent adverse events occurring in ≥5% of patients or with between-groups p <.10, mean changes in weight and certain laboratory parameters (i.e., fasting glucose, lipids, and prolactin measures), and treatment-emergent categorical changes in these laboratory values [10,11].

Efficacy measures included time to relapse and rate of relapse. Relapse was defined a priori as 1) an increase of any Brief Psychiatric Rating Scale (BPRS) [12] positive symptom item to a score >4, with an absolute increase ≥2 for the specific item since randomization; or 2) an increase of any BPRS positive symptom item to a score >4, with an absolute increase ≥4 on the positive symptom subscale since randomization; or 3) hospitalization as the result of worsening of positive psychotic symptoms. Other efficacy/effectiveness measures included mean change in Positive and Negative Syndrome Scale (PANSS) [13] total, positive, and negative scores; time to all-cause discontinuation; rate of overall discontinuation; and rate of discontinuation due to efficacy-related reasons. "Efficacy-related reasons" was defined as discontinuation due to lack of efficacy or discontinuation due to any *psychiatric *adverse event (e.g., "schizophrenia," "paranoid disorder," etc.).

### Procedures and Dosing

Outpatients with schizophrenia who had maintained stability on open-label oral olanzapine for 4 to 8 weeks were randomly assigned to receive 1 of the following olanzapine LAI dosages for 24 weeks: 1) 150 mg every 2 weeks, hereafter referred to as "low" (N = 140), 2) 405 mg every 4 weeks, hereafter referred to as "medium" (N = 318), or 3) 300 mg every 2 weeks, hereafter referred to as "high" (N = 141). The approximate oral equivalents for these olanzapine LAI dosages are 10 mg/day for the low dose, 15 mg/day for the medium dose, and 20 mg/day for the high dose.

### Statistical Analyses

The primary objective of these post hoc analyses was to examine the potential relationship between different olanzapine LAI doses and changes in safety and efficacy parameters. For continuous (or mean change) data, we used linear regression to analyze the relationship between dose and the endpoint assessments of PANSS total score, prolactin, weight, and fasting glucose and lipids measures. These endpoint assessments were the mean change from baseline using the last-observation-carried forward (LOCF) method. Patients were only included in the analysis if they had a baseline and at least one post-baseline assessment. To check the robustness of the analyses, we also utilized the non-parametric Jonckheere-Terpstra [14] test for the safety measures including mean changes in weight, as well as fasting glucose, lipids, and prolactin measures. Effect sizes between doses were estimated as the difference of the least-squares means divided by the square root of model residual variance. Least-squares estimates were obtained from the following ANOVA model: change from baseline = therapy + geographic region.

For categorical data, we used the Cochran-Armitage test [15] for incidence of treatment-emergent adverse events, treatment-emergent categorical changes in prolactin, weight, and fasting glucose and lipids (using American Diabetes Association and National Cholesterol Education Program criteria) [11,16], and relapse and discontinuation rates. For time-to-event measures, we used Kaplan-Meier survival method and compared potential dose effects using the log-rank test. Finally, we calculated number needed to treat (NNT) and number needed to harm (NNH) values for the categorical safety and efficacy outcomes.

Unless otherwise specified, analyses were performed on a subset of the original intent-to-treat population; that is, those patients in the 3 treatment groups (3 clinical olanzapine LAI doses) from the original study. Statistical significance was defined as 2-tailed p <.05. Because the purpose of the analyses was to detect a potential relationship between changes in certain efficacy and safety variables and 3 different study drug doses, no type I error adjustments for multiplicity were performed.

## Results

### Safety and Tolerability Analyses

As reported in the published manuscript [2], the 3 dose groups did not significantly differ on any baseline demographic or illness characteristics, with the exception of mean baseline PANSS total score (high-dose group significantly higher at baseline than the medium-dose group, 56.8 vs. 55.1).

Table 1 presents the incidence and the Cochran-Armitage trend test p-values for treatment-emergent adverse events occurring in ≥5% of patients in the dose groups, or with between-groups p <.10. Only "increased appetite" showed a significant dose association, the incidence of which increased with increasing dose.

**Table 1:** Treatment-emergent adverse events occurring in ≥5% of patients or with between-groups p <.10

	OLZ LAI 150 (N = 140)	OLZ LAI 405 (N = 318)	OLZ LAI 300 (N = 141)	**Overall p-value**^**a**^	Cochran-Armitage Test p-value
**Anxiety**	5 (3.57%)	17 (5.35%)	7 (4.96%)	.767	.686
**Headache**	7 (5.00%)	9 (2.83%)	3 (2.13%)	.355	.216
**Increased appetite**	1 (0.71%)	3 (0.94%)	5 (3.55%)	.080	.031
**Insomnia**	11 (7.86%)	23 (7.23%)	9 (6.38%)	.871	.632
**Nasopharyngitis**	8 (5.71%)	11 (3.46%)	7 (4.96%)	.442	.947
**Schizophrenia**	6 (4.29%)	3 (0.94%)	2 (1.42%)	.047	.165
**Somnolence**	8 (5.71%)	10 (3.14%)	5 (3.55%)	.367	.467
**Weight increased**	12 (8.57%)	16 (5.03%)	15 (10.64%)	.071	.267

Abbreviations: OLZ LAI = olanzapine long-acting injection; OLZ LAI 150 = group receiving low-dose olanzapine LAI, 150 mg every 2 weeks, approximate oral equivalent 10 mg/day (N = 140); OLZ LAI 405 = group receiving medium-dose olanzapine LAI, 405 mg every 4 weeks, approximate oral equivalent 15 mg/day (N = 318); and OLZ LAI 300 = group receiving high-dose olanzapine LAI, 300 mg every 2 weeks, approximate oral equivalent 20 mg/day (N = 141).

^a^Test of overall group differences based on Fisher's exact test.

**Figure 1:**
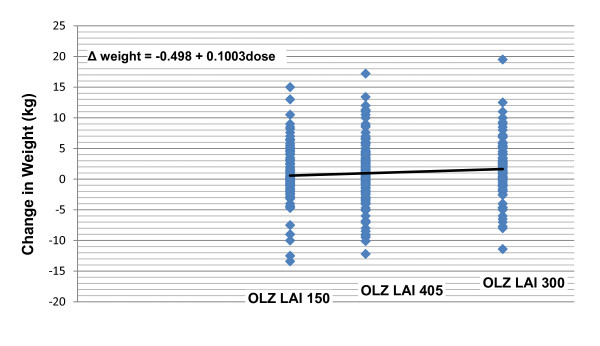
**Regression scatterplot of mean change in weight at endpoint (LOCF) by dose**. Figure 1 shows the scatterplot, regression line, and resulting equation for the relationship between endpoint weight change and dose. In the equation, "dose" refers to the calculated oral equivalent daily dose (10.7, 14.5, or 21.4 mg/day), not the actual injected dose. Abbreviations: OLZ LAI = olanzapine long-acting injection; OLZ LAI 150 = group receiving low-dose olanzapine LAI, 150 mg/2 weeks, approximate oral equivalent 10 mg/day (N = 140); OLZ LAI 405 = group receiving medium-dose olanzapine LAI, 405 mg/4 weeks, approximate oral equivalent 15 mg/day (N = 318); and OLZ LAI 300 = group receiving high-dose olanzapine LAI, 300 mg/2 weeks, approximate oral equivalent 20 mg/day (N = 141).

**Figure 2:**
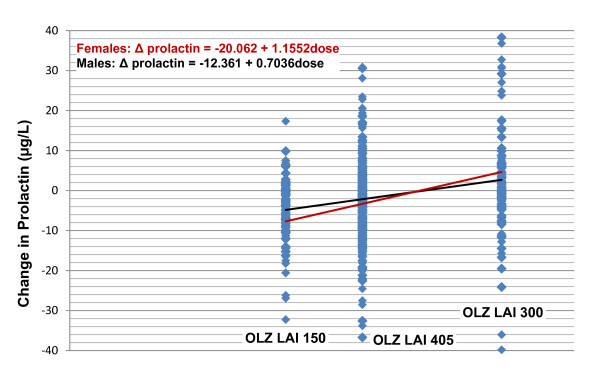
**Regression scatterplots of mean change in prolactin at endpoint (LOCF) by dose and gender**. Figure 2 shows the scatterplots, regression lines, and resulting equations for the relationship between endpoint prolactin change and dose, by gender. In the equation, "dose" refers to the calculated oral equivalent daily dose (10.7, 14.5, or 21.4 mg/day), not the actual injected dose. Abbreviations: OLZ LAI = olanzapine long-acting injection; OLZ LAI 150 = group receiving low-dose olanzapine LAI, 150 mg/2 weeks, approximate oral equivalent 10 mg/day (N = 140); OLZ LAI 405 = group receiving medium-dose olanzapine LAI, 405 mg/4 weeks, approximate oral equivalent 15 mg/day (N = 318); and OLZ LAI 300 = group receiving high-dose olanzapine LAI, 300 mg/2 weeks, approximate oral equivalent 20 mg/day (N = 141).

Table 2 provides the mean changes in weight and in several fasting glucose, lipids, and prolactin laboratory measures, along with results of the trend tests from both a regression analysis and the Jonckheere-Terpstra test. Significant dose-associated changes were identified for weight and for prolactin, both of which exhibited mean increases with increasing dose. The resulting scatterplots, trendlines, and regression equations are shown in Figure 1 for weight and in Figure 2 for prolactin. Note that in these equations, "dose" refers to the calculated oral equivalent daily dose (i.e., 10.7, 14.5, or 21.4 mg/day), not the actual injected dose.

**Table 2:** Mean changes in weight and laboratory values

	OLZ LAI 150 (N = 140) Mean (SD)	OLZ LAI 405 (N = 318) Mean (SD)	OLZ LAI 300 (N = 141) Mean (SD)	R-Square	Regression Slope	Regression p-value	Jonckheere-Terpstra p-value	Effect Sizes		
								
								High vs. Low (95% C.I.)	Medium vs. Low (95% C.I.)	High vs. Medium (95% C.I.)
**Weight (kg)**	0.67 (4.38)	0.89 (3.87)	1.70 (4.14)	0.0086	0.10	.024	.034	0.264	0.063	0.201
**in lbs:**	1.47 (9.64)	1.96 (8.51)	3.74 (9.11)							
**Fasting glucose (mmol/L)**	0.14 (1.36)	0.17 (1.29)	0.22 (1.19)	0.0005	0.01	.642	.092	0.049	0.016	0.033
**in mg/dL**:	2.52 (24.50)	3.06 (23.24)	3.96 (21.44)							
**Fasting HDL (mmol/L)**	-0.00 (0.22)	0.00 (0.24)	-0.05 (0.24)	0.0064	-0.01	.094	.215	0.196	0.022	0.218
**in mg/dL**:	-0.00 (8.49)	0.00 (9.27)	-1.93 (9.27)							
**Fasting LDL (mmol/L)**	-0.04 (0.57)	-0.07 (0.64)	0.02 (0.73)	0.0015	0.01	.423	.169	0.078	0.041	0.119
**in mg/dL**:	-1.54 (22.01)	-2.70 (24.71)	0.77 (28.19)							
**Fasting total cholesterol (mmol/L)**	-0.12 (0.65)	-0.07 (0.73)	0.01 (0.78)	0.0037	0.01	.199	.240	0.165	0.065	0.100
**in mg/dL**:	-4.63 (25.10)	-2.70 (28.19)	0.39 (30.12)							
**Fasting triglycerides (mmol/L)**	-0.18 (1.84)	-0.03 (1.23)	0.03 (1.19)	0.0022	0.02	.324	.958	0.147	0.107	0.039
**in mg/dL**:	-15.93 (162.83)	-2.65 (108.85)	2.65 (105.31)							
**Prolactin (μg/L)**:										
**All patients**	-5.61 (12.49)	-2.76 (19.02)	3.58 (33.78)	0.0209	0.87	.001	<.001	0.410	0.127	0.283
**Females**	-8.27 (16.1)	-2.86 (30.31)	4.33 (23.16)	0.0294	1.16	.026	<.001	0.493	0.212	0.281
**Males**	-3.88 (9.17)	-2.72 (9.65)	3.16 (38.56)	0.0159	0.70	.025	.018	0.347	0.047	0.300

Abbreviations: OLZ LAI = olanzapine long-acting injection; OLZ LAI 150 = group receiving low-dose olanzapine LAI, 150 mg every 2 weeks, approximate oral equivalent 10 mg/day (N = 140); OLZ LAI 405 = group receiving medium-dose olanzapine LAI, 405 mg every 4 weeks, approximate oral equivalent 15 mg/day (N = 318); and OLZ LAI 300 = group receiving high-dose olanzapine LAI, 300 mg every 2 weeks, approximate oral equivalent 20 mg/day (N = 141); HDL = high-density lipoprotein cholesterol; LDL = low-density lipoprotein cholesterol.

Table 3 presents the incidence of categorical changes in these laboratory measures at endpoint, along with results of the Cochran-Armitage trend tests. Significant dose associations were identified for fasting high-density lipoprotein (HDL) cholesterol normal at baseline to low at endpoint and fasting triglycerides normal at baseline to high at endpoint, the incidence of which increased with increasing dose.

**Table 3:** Categorical changes in weight and laboratory values at endpoint

		OLZ LAI 150 n/N (%)	OLZ LAI 405 n/N (%)	OLZ LAI 300 n/N (%)	Cochran-Armitage p-value
**Weight**	≥7% gain	17/140 (12.14)	34/315 (10.79)	24/140 (17.14)	.127
	≥7% loss	9/140 (6.43)	13/315 (4.13)	7/140 (5.00)	.720
**Fasting glucose**	Normal to borderline	14/75 (18.67)	37/176 (21.02)	25/89 (28.09)	.121
	Normal/borderline to high	5/106 (4.72)	6/244 (2.46)	4/108 (3.70)	.855
	Normal to high	1/75 (1.33)	2/176 (1.14)	0/89 (0)	.311
	Borderline to high	4/31 (12.90)	4/68 (5.88)	4/19 (21.05)	.320
**Fasting HDL**	Normal to low	8/58 (13.79)	26/133 (19.55)	19/62 (30.65)	.019
**Fasting LDL**	Normal to borderline	6/26 (23.08)	15/66 (22.73)	5/18 (27.78)	.702
	Normal to high	0/26 (0)	0/66 (0)	0/18 (0)	-------
	Borderline to high	6/53 (11.32)	14/129 (10.85)	9/73 (12.33)	.804
**Fasting total cholesterol**	Normal to borderline	9/48 (18.75)	15/133 (11.28)	10/51 (19.61)	.607
	Normal to high	0/48 (0)	2/133 (1.50)	2/51 (3.92)	.127
	Borderline to high	4/34 (11.76)	13/65 (20.00)	5/35 (14.29)	.959
**Fasting triglycerides**	Normal to borderline	13/62 (20.97)	17/133 (12.78)	9/53 (16.98)	.713
	Normal to high	2/62 (3.23)	8/133 (6.02)	10/53 (18.87)	.001
	Normal to extremely high	0/62 (0)	0/133 (0)	1/53 (1.89)	.082
	Borderline to high	3/14 (21.43)	11/38 (28.95)	3/18 (16.67)	.556
	Borderline to extremely high	0/14 (0)	0/38 (0)	1/18 (5.56)	.114
**Prolactin**	Normal to high (females)	2/17 (11.76)	9/42 (21.43)	7/21 (33.33)	.112
	Normal to high (males)	4/49 (8.16)	16/116 (13.79)	7/52 (13.46)	.523

Abbreviations: OLZ LAI = olanzapine long-acting injection; OLZ LAI 150 = group receiving low-dose olanzapine LAI, 150 mg every 2 weeks, approximate oral equivalent 10 mg/day (N = 140); OLZ LAI 405 = group receiving medium-dose olanzapine LAI, 405 mg every 4 weeks, approximate oral equivalent 15 mg/day (N = 318); and OLZ LAI 300 = group receiving high-dose olanzapine LAI, 300 mg every 2 weeks, approximate oral equivalent 20 mg/day (N = 141); HDL = high-density lipoprotein; LDL = low-density lipoprotein.

### Efficacy and Effectiveness Analyses

**Figure 3:**
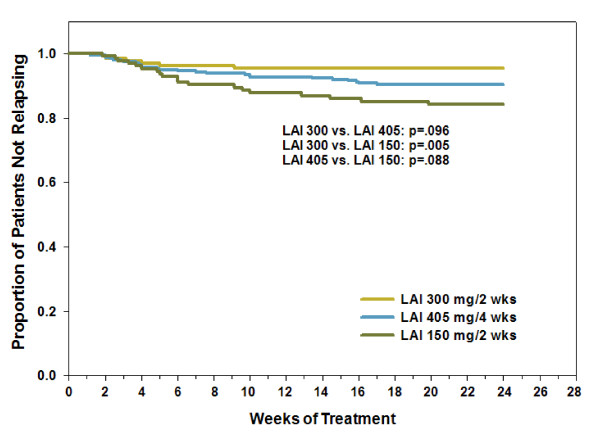
**Kaplan-Meier survival analysis of time to relapse by dose group**. Figure 3 illustrates the survival curves for time to relapse by dose group. Relapse or "psychotic exacerbation" was defined as 1) an increase of any BPRS positive symptom item to a score >4, with an absolute increase ≥2 for the specific item since randomization, 2) an increase of any BPRS positive symptom item to a score >4, with an absolute increase ≥4 on the positive symptom subscale since randomization; or 3) hospitalization as the result of worsening of positive psychotic symptoms. Median time to relapse not reported because no group had >50% rate of relapse. Abbreviations: OLZ LAI = olanzapine long-acting injection; OLZ LAI 150 = group receiving low-dose olanzapine LAI, 150 mg every 2 weeks, approximate oral equivalent 10 mg/day (N = 140); OLZ LAI 405 = group receiving medium-dose olanzapine LAI, 405 mg every 4 weeks, approximate oral equivalent 15 mg/day (N = 318); and OLZ LAI 300 = group receiving high-dose olanzapine LAI, 300 mg every 2 weeks, approximate oral equivalent 20 mg/day (N = 141).

Figure 3 presents the Kaplan-Meier survival curves by dose group for time to relapse. The high-dose group had a significantly longer time to relapse than the low-dose group. Rates of relapse (low: 16%, medium: 10%, high: 5%) also showed a significant dose association based on the Cochrane-Armitage test (p = .003), indicating declining relapse rates with increasing dose.

**Figure 4:**
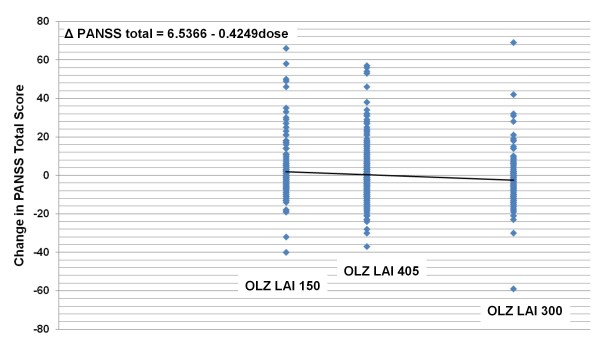
**Regression scatterplot of mean change in PANSS total score (LOCF) by dose**. Figure 4 shows the scatterplot, regression line, and resulting equation for the relationship between endpoint PANSS total score change and dose (R^2 ^= 0.0133). In the equation, "dose" refers to the calculated oral equivalent daily dose (10.7, 14.5, or 21.4 mg/day), not the actual injected dose. Abbreviations: OLZ LAI = olanzapine long-acting injection; OLZ LAI 150 = group receiving low-dose olanzapine LAI, 150 mg every 2 weeks, approximate oral equivalent 10 mg/day (N = 140); OLZ LAI 405 = group receiving medium-dose olanzapine LAI, 405 mg every 4 weeks, approximate oral equivalent 15 mg/day (N = 318); and OLZ LAI 300 = group receiving high-dose olanzapine LAI, 300 mg every 2 weeks, approximate oral equivalent 20 mg/day (N = 141).

A statistically significant dose association based on regression analysis was identified for baseline-to-endpoint mean change in PANSS total score (low: +2.66 [SD = 14.95], medium: -0.09 [SD = 13.47], high: -2.19 [SD = 13.11], p <.01) and PANSS positive score (p = .04), but not PANSS negative score (p = .08). The resulting scatterplot, trendline, and regression equation are shown in Figure 4. Mean PANSS total scores declined with increasing dose, indicated by the negative slope. Effect sizes for PANSS total were: 0.356 high vs. low, 0.203 medium vs. low, and 0.152 high vs. medium. Effect sizes for PANSS positive were 0.258 high vs. low, 0.167 medium vs. low, and 0.091 high vs. medium.

**Figure 5:**
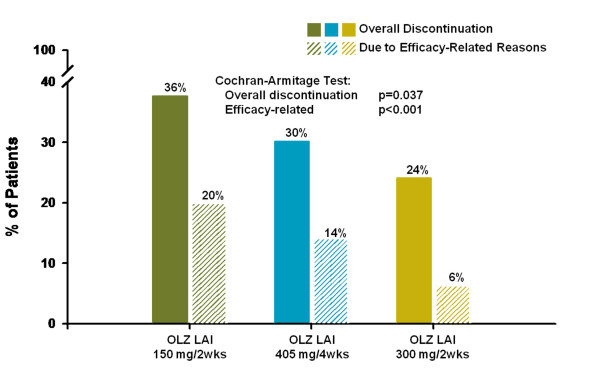
**Rates of overall discontinuation and discontinuation due to efficacy-related reasons by dose group**. Figure 5 illustrates the rates of overall discontinuation (NNT = 9 high vs. low dose) and discontinuation for efficacy-related reasons (NNT = 8 high vs. low dose; NNT = 13 high vs. medium dose) by dose group. Efficacy-related reasons includes lack of efficacy and/or any *psychiatric *adverse event (e.g., "schizophrenia", "paranoid disorder," etc.). Abbreviations: OLZ LAI = olanzapine long-acting injection; OLZ LAI 150 = group receiving low-dose olanzapine LAI, 150 mg every 2 weeks, approximate oral equivalent 10 mg/day (N = 140); OLZ LAI 405 = group receiving medium-dose olanzapine LAI, 405 mg every 4 weeks, approximate oral equivalent 15 mg/day (N = 318); and OLZ LAI 300 = group receiving high-dose olanzapine LAI, 300 mg every 2 weeks, approximate oral equivalent 20 mg/day (N = 141).

**Figure 6:**
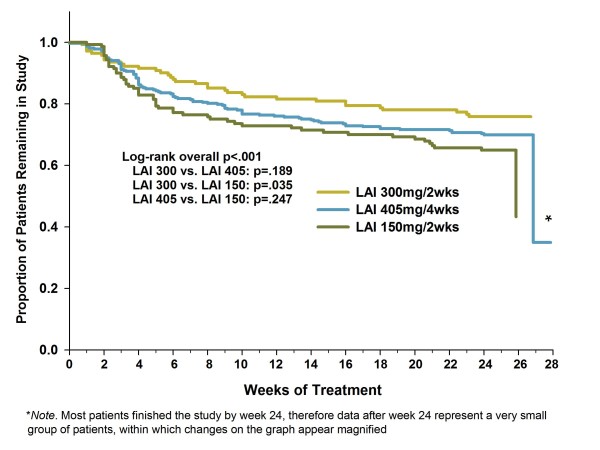
**Kaplan-Meier survival analysis of time to all-cause discontinuation by dose group**. Figure 6 shows the survival curves for time to all-cause discontinuation by dose group. Abbreviations: OLZ LAI = olanzapine long-acting injection; OLZ LAI 150 = group receiving low-dose olanzapine LAI, 150 mg every 2 weeks, approximate oral equivalent 10 mg/day (N = 140); OLZ LAI 405 = group receiving medium-dose olanzapine LAI, 405 mg every 4 weeks, approximate oral equivalent 15 mg/day (N = 318); and OLZ LAI 300 = group receiving high-dose olanzapine LAI, 300 mg every 2 weeks, approximate oral equivalent 20 mg/day (N = 141).

Figure 5 presents discontinuation rates, both overall (low: 36%, medium: 30%, high: 24%, p = .037) and due to efficacy-related reasons (low: 20%, medium: 14%, high: 6%, p <.001). Significant dose associations were identified for both of these measures based on the Cochran-Armitage test, indicating declining discontinuation rates with increasing dose. Figure 6 presents the Kaplan-Meier survival curves by dose group for time to all-cause discontinuation. The high-dose group had a significantly longer time to all-cause discontinuation than the low-dose group.

### Number Needed to Treat (NNT)/Number Needed to Harm (NNH) Analyses

For changes in HDL cholesterol normal at baseline to low at endpoint, the NNH was 6 (95% CI: 4 to 43) when comparing high- and low-dose groups. This NNH value indicates that for every 6 patients treated with the high dose instead of the low dose for 24 weeks, 1 additional HDL change (normal to low) can be expected. For changes in triglycerides from normal at baseline to high at endpoint, the NNH was 8 (95% CI: 5 to 64) when comparing the high-dose with the medium-dose group and 7 (95% CI: 4 to 24) when comparing the high-dose with the low-dose group. These NNH values indicate that 1 additional triglycerides change (normal to high) can be expected to occur for every 8 patients treated with the high dose instead of the medium dose, or for every 7 patients treated with the high dose instead of the low dose.

The NNT for relapse rate was 20 (95% CI: 11 to 294) when comparing the high-dose with the medium-dose group and 9 (95% CI: 6 to 24) when comparing the high-dose with the low-dose group. These NNT values indicate that 1 less relapse can be expected for every 20 patients treated with the high dose instead of the medium dose for 24 weeks; likewise, 1 less relapse can be expected for every 9 patients treated with the high dose instead of the low dose. As with relapse rate, the NNT for overall discontinuation rate comparing high dose and low dose was 9 (95% CI: 5 to 103), indicating that for every 9 patients treated with the high dose instead of the low dose, 1 additional patient is expected not to discontinue treatment. The NNT for discontinuation due to efficacy-related reasons was 8 (95% CI: 5 to 18) for high versus low dose and 13 (95% CI: 8 to 46) for high versus medium dose.

Additional variables analyzed for NNT or NNH were not significant.

## Discussion

These analyses further investigated apparent dose-associated changes for both safety and efficacy variables observed in a 24-week maintenance study of olanzapine LAI in patients with schizophrenia [2]. In this post hoc analysis, we identified statistically significant dose-associated changes for several measures, including safety/tolerability variables (incidence of increased appetite, mean changes in weight and prolactin, categorical changes in HDL cholesterol and triglycerides) and efficacy/effectiveness measures (i.e., relapse, PANSS change, and discontinuation). When making a decision about the dosage of olanzapine LAI, both safety and efficacy must be considered, along with other factors. This analysis suggests that higher doses of olanzapine LAI may be associated with both clinically desirable efficacy outcomes, including fewer relapses and longer time to discontinuation, and disadvantageous changes in certain safety outcomes, such as greater weight gain.

Prior studies have inconsistently found dose-associated changes in safety or efficacy measures for oral olanzapine. Several articles have reported a lack of dose-associated changes for oral olanzapine within the labeled dose range [4,17,18]; however, others have identified differences in safety and/or efficacy changes between doses within the labeled dose range and a higher dose which appear to be related to olanzapine plasma concentrations [3,5]. Why did the current analysis find a dose relationship? The true fixed-dose design of the study used for this analysis was more suited for a dose comparison than were some previous oral olanzapine studies, which allowed for small dose adjustments (increases and decreases) within the fixed dose designs. Additionally, the nature of the injection itself provided for a more controlled dose comparison. Olanzapine LAI delivers a continuous, consistent dose of olanzapine to the system, so any variations due to inconsistent medication adherence, intentional or otherwise, are eliminated, allowing for a closer link between prescribed dose and systemic concentrations. While plausible hypotheses, additional research is needed to verify these findings in other studies and to explore related factors. Experience in clinical practice, outside of registration trials, may also provide needed perspective.

The NNT and NNH analyses provide important clinical insight into selecting doses. There were both advantages and disadvantages to using the highest dose rather than the lowest dose, and also for using the highest dose versus the medium dose. We did not find significant NNT or NNH values comparing low dose with medium dose, suggesting that any clinical differences between these 2 doses could be difficult to detect in practice. It should be noted that in addition to the 3 doses described here, there are 2 other doses of olanzapine LAI that have been studied (300 mg every 4 weeks and 210 mg every 2 weeks, which are roughly equivalent to 150 mg every 2 weeks and 405 mg every 4 weeks, respectively). In addition, the doses described in this analysis are for maintenance treatment; prescribers should refer to the medication labeling for recommendations on initiating therapy with olanzapine LAI.

There are some important limitations to the current analyses. First, these analyses were conducted post hoc in a single clinical trial and therefore should be considered exploratory. Next, it is important to note that because this was a maintenance study, patients in this analysis had been taking open-label oral olanzapine for 4 to 8 weeks prior to baseline to establish clinical stability; thus, any adverse events occurring during that lead-in phase are not represented here (although lead-in phase data were collected and are reported in the primary manuscript [2]). Therefore, certain safety signals, such as weight gain or metabolic changes, may be underestimated in this analysis. At the same time, as we did not correct for multiple comparisons, some safety signals could be false positives occurring due to chance alone. Another consideration is that the very low dose of olanzapine LAI, 45 mg/4 weeks (approximately 1.6 mg/day oral equivalent) was not included. Whereas inclusion of this very low dose would have allowed an examination of trends across a much wider dosing range, this dose was shown to be non-therapeutic in the study [2], is unlikely to be utilized in clinical practice, and could lead to a distortion of the trends in the true dosing range of olanzapine LAI. Finally, one aspect of the study design affects how safety and efficacy data are interpreted; specifically, some patients experienced a change in dose from the stabilization period to the maintenance period. Patients who had been stabilized on 20 mg/day oral olanzapine and subsequently randomized to the lowest olanzapine LAI dose (approximately 10 mg/day oral equivalent) essentially experienced a dose decrease. Likewise, patients stabilized on 10 mg/day oral olanzapine and subsequently randomized to the highest olanzapine LAI dose (approximately 20 mg/day oral equivalent) experienced a dose increase. These dose changes make interpreting our findings regarding safety and efficacy more difficult.

## Conclusions

Analyses of several clinically important safety and efficacy measures revealed significant dose-associated changes for olanzapine LAI, with the highest dose generally showing greater efficacy (e.g., longer time to relapse) as well as greater changes in certain safety measures (e.g., greater weight gain). When considering olanzapine LAI, as with all antipsychotics, it is important to carefully consider the potential benefits and risks for an individual patient.

## Competing interests

All coauthors are employees and/or shareholders of Eli Lilly and Company.

## Authors' contributions

ALH was responsible for proposing the project and designing the analyses. BS was responsible for designing as well as conducting the statistical analyses. JLK was responsible for designing the analyses. SBW was responsible for the literature review and for drafting and revising the manuscript. DPM provided medical leadership and was responsible for data collection and design of the original study. In addition to these roles, all coauthors contributed to the interpretation of the results and reviewed and approved the final version of the manuscript.

## Pre-publication history

The pre-publication history for this paper can be accessed here:

http://www.biomedcentral.com/1471-244X/11/28/prepub
